# Supplementary material for: Meteorological drivers of resource adequacy failures in current and high renewable Western U.S. power systems
Source: Nat Commun. 2023 Oct 11;14:6379. doi: 10.1038/s41467-023-41875-6 (PMC10567759; doi:10.1038/s41467-023-41875-6)
Supplement: Supplementary file 1 — Supplementary Information [file 41467_2023_41875_MOESM1_ESM.pdf]

Supplementary Information for Meteorological Drivers of Resource Adequacy Failures in Current and High Renewable Western U.S. Power Systems

June 25, 2023

1 Capacity factors

1.1 Solar

We derive hourly solar capacity factors for a EFG-Polycrystalline silicon photovoltaic module as[1]:

$$CF_{pv}^{t} = P_{R}^{t}\frac{RSDS^{t}}{RSDS_{STC}} \tag{A.1}$$

where $RSDS^t$ hourly represents surface downwelling shortwave flux in air $[Wm^{-2}]$ for which we use the *surface solar radiation downwards* variable from ERA5, and the superscript t indexes the hour. Though the variable is referred with short name *SSRD* in ERA5 datasets, we refer to it as *RSDS* following the CF conventions used in climate model intercomparison projects (CMIP) and in various literature. In ERA5 data, this quantity is captured as hourly energy accumulation with units Jm^{-2} but we need to calculate power derived from solar radiation, so we divide hourly accumulation by $3600s$ to obtain the average power during the hour with units Wm^{-2} [1]. All the metorological variables are discreet in time and space (at the dataset resolution), and the index t is dropped hereafter for conciseness. In eq.A.1, $RSDS_{STC}$ refers to RSDS at standard test conditions and is equal to $1000Wm^{-2}$, and P_R^t is the hourly performance ratio calculated using

$$P_R = 1 + \gamma[T_{cell} - T_{STC}] \tag{A.2}$$

$$T_{cell} = c_1 + c_2TAS + c_3RSDS + c_4SWS \tag{A.3}$$

where T_{cell} is the PV cell temperature, TAS is surface air temperature (*2m temperature* in ERA5, converted from K to °C), and SWS is surface wind speed (calculated from *10m u- and v- components of wind* from ERA5). In eq.A.2, $\gamma = -0.005°C^{-1}$ and $T_{STC} = 25°C$. In eq.A.3, $c_1 = 4.3°C,\ c_2 = 0.943,\ c_3 = 0.028°Cm^2W^{-1}$, and $c_4 = -1.528°Csm^{-1}$ [2].

1.2 Wind

We calculate wind capacity factors using the formulation described in [3] for the composite 1.5 MW IEC class III turbine with power curves from the System Advisor Model (SAM) [4] as:

$$CF_{wind}^{t} = p(W_{100}^{t}) \tag{A.4}$$

where p is a function describing the power curve and W_{100}^t is the hourly corrected 100m wind speed. The correction accounts for air density and humidity related effects on the wind turbine performance and is carried out as:

$$W_{100} = W_{100,raw}\left(\frac{\rho_m}{1.225}\right)^{1/3} \tag{A.5}$$

$$\rho_m = \rho_d\left(\frac{1 + HUSS}{1 + 1.609 \times HUSS}\right) \tag{A.6}$$

$$\rho_d = \frac{PS}{\boldsymbol{R} \times (TAS + 273.15)} \tag{A.7}$$

[1] https://apps.ecmwf.int/codes/grib/param-db/?id=169

Eq.A.5 scales the wind speed $W_{100,raw}$ for air density as this affects the force exerted on the turbine blades, where ρ_m is the humidity corrected air density, which is in turn derived from the surface specific humidity ($HUSS$) as shown in eq.A.6. ρ_d is the dry air density which is derived using the ideal gas law from surface pressure [units-Pa](PS) and surface temperature (TAS) as shown in eq.A.7, where $\boldsymbol{R} = 287.058 Jkg^{-1}K^{-1}$ is the gas constant. $W_{100,raw}$ is calculated from the *100m u- and v- components of wind* from ERA5 data. Since ERA5 doesn't provide $HUSS$, we calculate it as (ref.[5]):

$$HUSS = \frac{0.622 \times VP}{0.01 \times PS - 0.378 \times VP} \tag{A.8}$$

$$VP = 6.112\, exp\left(\frac{17.67 \times TDPS}{TDPS + 243.5}\right) \tag{A.9}$$

where VP is the vapor pressure and $TDPS$ is the dewpoint temperature at surface in $^{\circ}C$(*2m temperature dewpoint temperature* in ERA5, converted from K to $^{\circ}C$).

Across WECC, few locations have wind speeds suitable for class I and II wind turbines based on the average wind speed over 2015-2020 from the ERA5 data (figure A.1).As a result, we estimate wind generation for all locations across WECC assuming a class-III wind turbine. The power curve from SAM is provided as the power output at discrete wind speeds (figure A.2), and we convert this into a continuous function through linear interpolation using the interp1d function from the SciPy package [cite]. We include the discrete power curve in this SI.

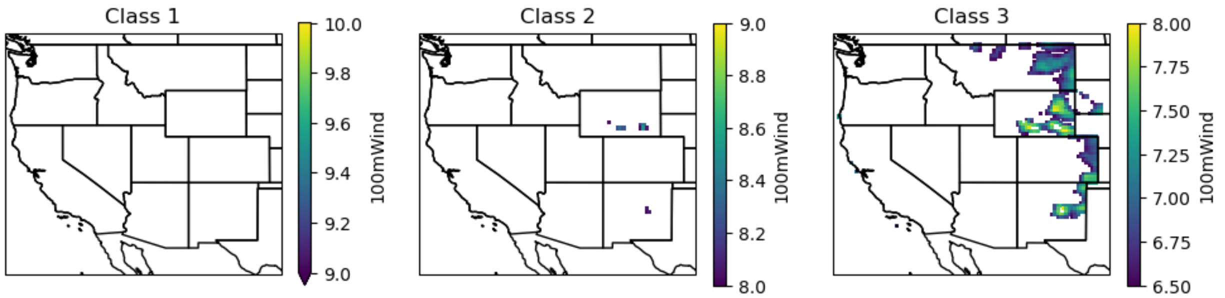

Figure A.1: Classification of geographical locations according to wind speed classes, based on 2015-2020 mean of 100m wind speeds

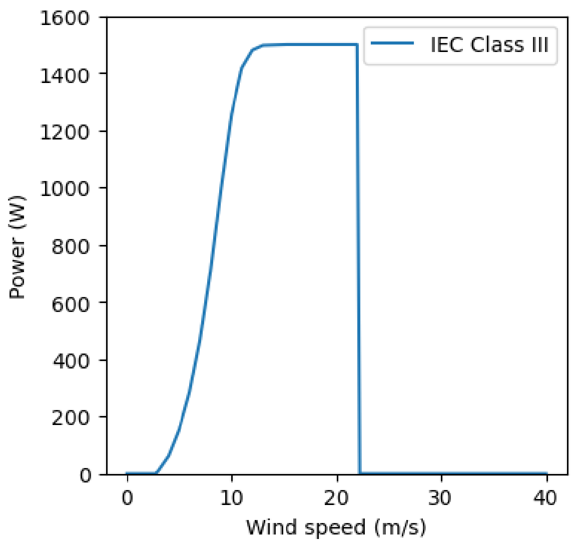

Figure A.2: Power curve for 1.5 MW IEC class III turbine

2 Capacity Expansion Model

The capacity expansion (CE) model optimizes new capacity investments, operations of new and existing units, and inter-regional electricity transfers by minimizing total system costs subject to system and unit-level constraints. Total system costs equal the sum of the cost of electricity generation of existing and new units and the

Parameter	Definition	Unit
P_c^{MAX}	Maximum power rating of new unit c	MW
$P_{c_s}^{EMAX}$	Maximum energy capacity of new storage unit c_s	MW
P_l^{MAX}	Maximum transmission capacity of line l	MW
FOM_c	Fixed O&M cost of new unit c	$/MW/year
OCC_c	Overnight capital cost of new unit c	$/MW
OCC_l	Overnight capital cost of transmission expansion along line l	$/MW
CRF_c	Capital recovery factor of new unit c	$/MW
CRF_l	Capital recovery factor of new transmission line l	$/MW
OC_c	Operational cost of new unit c	$/MWh
VOM_c	Variable O&M cost of new unit c	$/MWh
VOM_i	Variable O&M cost of existing unit i	$/MWh
OC_i	Operational cost of existing unit i	$/MWh
OC_c	Operational cost of new unit c	$/MWh
FC_c	Fuel cost of new unit c	$/MMBtu
FC_i	Fuel cost of existing unit i	$/MMBtu
HR_c	Heat rate of new unit c	MMBtu/MWh
HR_i	Heat rate of existing unit i	MMBtu/MWh
R	Discount rate = 0.07	–
LT_c	Life time of new units c	Years
N_c^{MAX}	Maximum number of new renewable units c built	Whole number
M	Planning reserve margin as fraction of peak demand	–
$D_{z,t}$	Total load (or electricity demand) in region z at time t	MWh
D_t	Total load (or electricity demand) across regions at time t	MWh

Table A.1: List of Parameters

cost of new capacity investments. Electricity generation costs equal the sum of fixed operations and maintenance (O&M) costs and variable electricity generation costs, which include fuel costs and variable O&M costs. The model runs till year 2030 in a 8 year increment to meet the prescribed renewable electricity (RE) penetration level for the US Western Interconnection (WECC). In each time step, the CE model can add any number of coal steam with carbon capture and sequestration (CCS), natural gas combined cycle (NGCC), NGCC with CCS, nuclear, wind, solar generators, battery and long-duration storage units, as well as DAC units and transmission line capacities.

2.1 Functional Forms

2.1.1 Parameters and Variables

Parameter	Definition	Unit
$P_{t,z}^{MAX,WIND}$	Maximum aggregate wind profile in region z at time t	MW
$P_{t,z}^{MAX,SOLAR}$	Maximum aggregate solar profile in region z at time t	MW
$H_{b,z}$	Maximum hydropower generation in region z and time block b	MWh
$Q_{i_s}^{MAX}$	Maximum charging rate of storage unit i_s	MW
$Q_{c_s}^{MAX}$	Maximum charging rate of new storage unit c_s	MW
$FOR_{i,t}$	Forced outage rate of existing unit i at time t	–
FOR_t^{RE}	Forced outage rate of existing wind and solar units at time t	–
$FOR_{c,t}$	Forced outage rate of new unit c at time t	–
RR	Renewable generation requirement as a fraction of total WECC-wide demand	–
$CF_{c_r,t}$	Capacity factor of new renewable unit c_r at time t	–
W_b	Scaling factor from number of representative to total hours in time block b	–
$X_{i_s}^{MAX}$	Maximum state of charge of existing storage unit i_s	MW
X_o	Initial state of charge as a fraction of maximum state of charge in each time block for existing and new storage units	–
RL_i	Maximum ramp rate of existing unit i	MW
RL_c	Maximum ramp rate of new unit c	MW
η	Round-trip efficiency of storage unit	%
ν	Transmission losses per unit of electricity transferred between regions	%

Table A.1: List of Parameters (Continued)

Set	Definition	Index	Note
$\mathbb{C}$	Set of potential new units	c	–
$\mathbb{C}_z$	Set of potential new units in region z	c_z	$\mathbb{C}_z \in \mathbb{C}$
$\mathbb{C}_r$	Set of potential new renewable units	c_r	$\mathbb{C}_r \in \mathbb{C}$
$\mathbb{C}_s$	Set of potential new storage units	c_s	$\mathbb{C}_s \in \mathbb{C}$
$\mathbb{C}_{s_z}$	Set of potential new storage units in region z	c_{s_z}	$\mathbb{C}_{s_z} \in \mathbb{C}_s$
$\mathbb{C}_{s'}$	Set of potential new non-storage units	$c_{s'}$	$\mathbb{C}_{s'} \in \mathbb{C}$
$\mathbb{I}$	Set of existing units	i	–
$\mathbb{I}_z$	Set of existing units in region z	i_z	$\mathbb{I}_z \in \mathbb{I}$
$\mathbb{I}_r$	Set of existing renewable units	i_r	$\mathbb{I}_r \in \mathbb{I}$
$\mathbb{I}_w$	Set of existing wind units	i_w	$\mathbb{I}_w \in \mathbb{I}$
$\mathbb{I}_{w_z}$	Set of existing wind units in region z	i_{w_z}	$\mathbb{I}_{w_z} \in \mathbb{I}_w$
$\mathbb{I}_o$	Set of existing solar units	i_o	$\mathbb{I}_o \in \mathbb{I}$
$\mathbb{I}_{o_z}$	Set of existing solar units in region z	i_{o_z}	$\mathbb{I}_{o_z} \in \mathbb{I}_o$
$\mathbb{I}_s$	Set of existing storage units	i_s	$\mathbb{I}_s \in \mathbb{I}$
$\mathbb{I}_{s_z}$	Set of existing storage units in region z	i_{s_z}	$\mathbb{I}_{s_z} \in \mathbb{I}_s$
$\mathbb{L}$	Set of transmission lines	l	–
$\mathbb{L}_z^{OUT}$	Set of transmission lines flowing out of region z	l_z^{OUT}	$\mathbb{L}_z^{OUT} \in \mathbb{L}$
$\mathbb{L}_z^{IN}$	Set of transmission lines flowing into region z	l_z^{IN}	$\mathbb{L}_z^{IN} \in \mathbb{L}$
$\mathbb{B}$	Set of time blocks	b	–
$\mathbb{T}$	Set of hours	t	–
$\mathbb{T}_p$	Set of peak demand hour	t_p	$\mathbb{T}_p \in \mathbb{T}$
$\mathbb{Z}$	Set of regions in WECC	z	–

Table A.2: List of Sets

Variable	Definition	Unit
n_c	Number of new units built of type c	Positive number
n_l	Total new transmission line capacity investments in line l	MW
k_{c_s}	Charge and discharge capacity built of new storage unit c_s	MW
e_{c_s}	State of charge capacity built of new storage unit c_s	MWh
$p_{i,t}$	Electricity generation (or electricity discharge) from existing unit i at time t	MWh
$p_{c,t}$	Electricity generation (or electricity discharge) from new unit c at time t	MWh
$f_{l,t}$	Total electricity flow in line l at time t	MWh
$q_{i_s,t}$	Electricity to charge existing storage unit i_s at time t	MWh
$q_{c_s,t}$	Electricity to charge new storage unit c_s at time t	MWh
$x_{i_s,t}$	State of charge of existing storage unit i_s at time t	MWh
$x_{c_s,t}$	State of charge of new storage unit c_s at time t	MWh

Table A.3: List of Variables

2.2 Objective Function

The CE model's objective function minimizes total annual fixed plus variable costs, where fixed costs capture investment costs in new transmission, electricity generators, and storage, and variable costs capture operational costs of new and existing generators:

$$\begin{aligned} TC^{CE} = & \left[\sum_{c_{s'}} n_{c_{s'}} \times P^{MAX}_{c_{s'}} \times \left(FOM_{c_{s'}} + OCC_{c_{s'}} \times CRF_{c_{s'}} \right) \right] \\ & + \left[\sum_{c_s} (k_{c_s} \times OCC_{c_s}) \times CRF_{c_s} \right] \\ & + \left[\sum_{l} n_l \times OCC_l \times CRF_l \right] + \left[\sum_{b} W_b \sum_{t_b \in T_b} \left(\sum_{c} p_{c,t_b} \times OC_c + \sum_{i} p_{i,t_b} \times OC_i \right) \right], \\ & \forall b \in \mathbb{B},\ i \in \mathbb{I},\ c \in \mathbb{C},\ c_{s'} \in \mathbb{C}_{s'},\ c_s \in \mathbb{C}_s, l \in \mathbb{L} \end{aligned} \tag{B.10}$$

where c indexes potential new units, including both non-storage and storage units; $c_{s'}$ indexes potential new non-storage units; c_s indexes potential new storage units; b indexes time blocks; t indexes time intervals (hours); i indexes existing units; l indexes potential new transmission lines; n_c is number of new unit investments; n_l is total new transmission line capacity investments in line l (MW); P^{MAX} is maximum capacity of unit (MW); FOM is fixed operation and maintenance (O&M) costs of units (\$/MW/year); OCC is overnight capital cost of new investments (\$/MW); CRF is capital recovery factor; k is power rating of new storage units; W is scaling factor from number of representative to total hours in time block; p_c is electricity generation from new unit c (MWh); p_i is electricity generation from existing unit i (MWh); and OC is operational costs of new or existing units (\$/MWh). OC is defined for new and existing generators as:

$$OC_i = VOM_i + HR_i \times FC_i \qquad \forall i \in \mathbb{I}, \tag{B.11a}$$

$$OC_c = VOM_c + HR_c \times FC_c \qquad \forall c \in \mathbb{C} \tag{B.11b}$$

where VOM is variable O&M costs (\$/MWh), HR is heat rate (MMBtu/MWh), and FC is fuel cost (\$/MMBtu). CRF_c is defined as:

$$CRF_c = \frac{R}{1 - \dfrac{1}{(1+R)^{LT_c}}} \qquad \forall c \in \mathbb{C}, \tag{B.12}$$

where R is discount rate and LT is plant lifetime (years).

2.3 System-level Constraints

The CE model enforces a planning reserve margin, which requires total adjusted capacity to exceed peak annual demand across WECC:

$$\begin{aligned} (1+M) \times D_t \quad \leq \quad & \sum_{c_t \in C_t} P^{MAX}_{c_t} \times FOR_{c_t,t} \times n_{c_t} \\ & + \sum_{c_r \in C_r} P^{MAX}_{c_r} \times FOR_{c_r,t} \times n_{c_r} \times CF_{c_r,t} \\ & + \sum_{c_s \in C_s} FOR_{c_s,t} \times k_{c_s} \\ & + \sum_{i \in (I - I_W - I_O)} FOR_{i,t} \times P^{MAX}_i \\ & + \sum_{z} \left(P^{MAX,SOLAR}_{z,t} + P^{MAX,WIND}_{z,t} \right) \times FOR^{RE}_t, \\ & \forall t \in \mathbb{T}_p \end{aligned} \tag{B.13}$$

where c_t and c_r index new thermal and renewable plant types, respectively; i_w and i_o index existing wind and solar generators, respectively; z indexes regions; M is a fraction of peak demand (equal to 0.13); FOR is forced outage rate; CF is capacity factor; $P^{MAX,SOLAR}$ is maximum regional generation by existing solar generators (MWh); $P^{MAX,WIND}$ is maximum regional generation by existing wind generators (MWh); and T_p indicates the annual peak demand hour. Adjusted capacity here accounts for temperature-dependent forced outage rates of generators [Table A.7] and hourly capacity factors for wind and solar facilities. Note that this PRM is enforced across all of WECC rather than on a region-by-region basis.

The CE model also requires supply balance demand at each time step:

$$\begin{aligned} D_{z,t} + \sum_{i_{s_z} \in \mathbb{I}_{s_z}} q_{i_{s_z},t} + \sum_{c_{s_z} \in \mathbb{C}_{s_z}} q_{c_{s_z},t} \qquad & \qquad \sum_{i_z \in \mathbb{I}_z} p_{i_z,t} + \sum_{c_z \in \mathbb{C}_z} p_{c_z,t} \\ + \sum_{l_z^{OUT} \in \mathbb{L}_z^{OUT}} f_{l_z^{OUT},t} \qquad \le & \qquad + \sum_{l_z^{IN} \in \mathbb{L}_z^{IN}} f_{l_z^{IN},t} \times \nu, \quad \forall z \in \mathbb{Z}, t \in \mathbb{T}, \end{aligned} \tag{B.14}$$

where z indexes zones, l indexes transmission lines, i_{s_z} indexes existing storage units in region z, c_{s_z} indexes new storage units in region z, i_z indexes existing units in region z, c_z indexes new units in region z, l_z^{IN} indexes lines flowing out of region z, l_z^{OUT} indexes transmission lines flowing out of region z, q is the electricity used to charge storage units (MWh), ν indicates losses for each unit of electricity imported into a region (assumed to be 5%), and f is electricity flows along transmission lines.

The total electricity flow through a transmission line ($f_{l,t}$) cannot exceed the line's initial transmission capacity (P_l^{MAX}) plus new capacity investments (n_l):

$$f_{l,t} \le P_l^{MAX} + n_l, \quad \forall l \in \mathbb{L},\ t \in \mathbb{T}, \tag{B.15}$$

where l indexes transmission lines, and $f_{l,t}$ is total electricity flow in line l at time t (MWh).

To examine power systems with increasing renewable penetrations, we constrain wind and solar generation to be greater than or equal to a percentage of total electricity demand:

$$\sum_{t,c_r} p_{c_r,t} + \sum_{t,i_r} p_{i_r,t} \ge \sum_{t,z} P_{z,t}^D \times RR, \quad \forall t \in \mathbb{T},\ c_r \in \mathbb{C}_r, i_r \in \mathbb{I}_r, z \in \mathbb{Z} \tag{B.16}$$

where RR equals the renewables requirement as a fraction of total demand. We enforce this constraint at the WECC-level.

2.4 Unit-level Constraints

2.4.1 Investment constraints

The CE model places an upper bound on wind and solar investments by grid cell based on the area of each grid cell and the energy density of wind and solar:

$$0 \le n_{c_r} \times P_{c_r}^{MAX} \le N_{c_r}^{MAX}, \quad \forall c_r \in \mathbb{C}_r \tag{B.17}$$

where n_{c_r} equals investment in new wind or solar plants. Maximum wind and solar investment per grid cell equals 8.8 and 55.5 GW, respectively, using densities of 0.9 and 5.7 W/m^2 [6] and the approximate area of 961 km^2 corresponding to a 0.25 Degree latitude x 0.25 Degree longitude grid cell.

2.4.2 Generation constraints

For existing generators, electricity generation is limited by the generators' capacities:

$$0 \le p_{i,t} \le P_i^{MAX}, \quad \forall t \in \mathbb{T},\ i \in \mathbb{I} \tag{B.18}$$

Combined electricity generation by existing wind and solar generators is limited to aggregate wind and solar generation profiles:

$$\sum_{i_{w_z} \in \mathbb{I}_{w_z}} p_{i_{w_z},t} \leq P_{z,t}^{MAX,WIND}, \quad \forall t \in \mathbb{T}, z \in \mathbb{Z}, \tag{B.19a}$$

$$\sum_{i_{o_z} \in \mathbb{I}_{o_z}} p_{i_{o_z},t} \leq P_{z,t}^{MAX,SOLAR}, \quad \forall t \in \mathbb{T}, z \in \mathbb{Z}, \tag{B.19b}$$

New generators' electricity generation cannot exceed their new capacity investments:

$$0 \leq p_{c,t} \leq n_c \times P_c^{MAX}, \quad \forall t \in \mathbb{T},\ c \in \mathbb{C} \tag{B.20}$$

Electricity generation by new renewable generators is also constrained by site-specific capacity factor time-series:

$$p_{c_r,t} \leq n_{c_r} \times P_{c_r}^{MAX} \times CF_{c_r,t}, \quad \forall t \in \mathbb{T},\ c_r \in \mathbb{C}_r \tag{B.21}$$

Hydropower generation is constrained based on observed data for each of our weather years. Since we ignore transmission constraints within each of our five regions, we aggregate hydropower capacity by region, then limit total hydropower generation by time block:

$$\sum_{t_b \in T_b, i_{h_z} \in I_{h_z}} p_{i_{h_z},t_b} \leq H_{b,z}, \forall z \in \mathbb{Z}, b \in \mathbb{B} \tag{B.22}$$

where i_{h_z} indexes all hydropower units in region z and $H_{b,z}$ equals maximum toal hydropower generation in time block b and region z [2.6.2].

The CE model places an upper bound on upwards changes in electricity generation from one time period to the next, i.e. in upward ramps, for new and existing units:

$$p_{i,t_b} - p_{i,t_b-1} \leq RL_i, \quad \forall t_b > 1, i \in \mathbb{I} \tag{B.23a}$$

$$p_{c,t_b} - p_{c,t_b-1} \leq n_c \times P_c^{MAX} \times RL_c \quad \forall t_b > 1, c \in \mathbb{C} \tag{B.23b}$$

where RL equals the ramp limit. We only constrain upwards ramps for two reasons: (1) downward ramps can be more easily achieved through curtailment of renewables than upwards ramps and (2) for computational tractability. Ramping constraints for new and existing generators are enforced between time periods within each time block, but not between time blocks.

2.4.3 Storage constraints

The energy capacity of storage built of (e_{c_s}) is constrained to a fixed energy to power ratio ($P_{c_s}^{EMAX}/P_{c_s}^{MAX}$) times invested power capacity:

$$0 \leq e_{c_s} \leq \frac{P_{c_s}^{EMAX}}{P_{c_s}^{MAX}} k_{c_s}, \quad \forall c_s \in \mathbb{C}_s \tag{B.24}$$

For storage units (i_s, c_s), state of charge (SOC) (x (MWh)) depends on the prior period's state of charge, electricity discharge (p (MWh)), and energy inflow (or charging) (q (MWh)) while accounting for round-trip efficiency (η) losses:

$$0 \leq x_{i_s,t} = x_{i_s,t-1} - 1/\sqrt{\eta} \times p_{i_s,t} + \sqrt{\eta} \times q_{i_s,t} \leq X_{i_s}^{MAX}, \quad \forall t > 1, i_s \in \mathbb{I}_s \tag{B.25a}$$

$$0 \leq x_{c_s,t} = x_{c_s,t-1} - 1/\sqrt{\eta} \times p_{c_s,t} + \sqrt{\eta} \times q_{c_s,t} \leq e_{c_s}, \quad \forall t > 1, c_s \in \mathbb{C}_s \tag{B.25b}$$

We assume 81% round-trip efficiency for all storage units.

In hour 1, the state of charge is assume to equal to a fixed fraction (X_o) of the maximum state of charge:

$$x_{i_s,t=1} = X_0 \times X_{i_s}^{MAX}, \quad \forall i_s \in \mathbb{I}_s \tag{B.26a}$$
$$x_{c_s,t=1} = X_0 \times e_{c_s}, \quad \forall c_s \in \mathbb{C}_s, \tag{B.26b}$$

where X_0 is the initial SOC fraction.

Charging and discharging are limited by max discharge and charge rates, which for new generators are decision variables noted above, and must be greater than zero:

$$p_{i_s,t} \leq P_{i_s}^{MAX}, \quad \forall i_s \in \mathbb{I}_s, t \in \mathbb{T} \tag{B.27a}$$
$$p_{c_s,t} \leq k_{i_s}, \quad \forall c_s \in \mathbb{C}_s, t \in \mathbb{T} \tag{B.27b}$$
$$0 \leq q_{i_s,t} \leq Q_{i_s}^{MAX}, \quad \forall i_s \in \mathbb{I}_s, t \in \mathbb{T} \tag{B.27c}$$
$$0 \leq q_{c_s,t} \leq k_{i_s}, \quad \forall c_s \in \mathbb{C}_s, t \in \mathbb{T} \tag{B.27d}$$

where $Q_{i_s}^{MAX}$ equals the maximum charging rate of storage assets, which we set equal to $P_{i_s}^{MAX}$.

Discharging cannot exceed the prior period's state of charge:

$$p_{i_s,t} \leq x_{i_s,t-1} \quad \forall i_s \in \mathbb{I}_s, t > 1 \tag{B.28a}$$
$$p_{c_s,t} \leq x_{c_s,t-1} \quad \forall c_s \in \mathbb{C}_s, t > 1 \tag{B.28b}$$

2.5 Model Solutions

The CE model solution determines new investments in generators, storage, and transmission assets by region or (in the case of wind and solar) grid cell; hourly electricity generation of new and existing units; hourly discharging, charging and states of charge of storage units; and electricity flows between regions. These solutions result from solving the optimization model described above with objective function B.10 subject to all constraints listed above [B.13,B.14,B.16,B.17,B.18,B.19,B.20,B.21,B.22,B.23a,B.24,B.25,B.26,B.27,B.28].

2.6 Data

In this section, we discuss the data and intermediate steps to calculate the parameters that are used in the model.

2.6.1 Regional Demand for Electricity

The sub-regional loads are constructed by aggregating loads in smaller balancing authorities located within their boundaries. Table

Sub-region	Balancing Authorities aggregated to find demand
CAMX	CISO, BANC, TIDC, LDWP
Desert Southwest	IID, AZPS, SRP, EPE, PNM, TEPC, WALC
NWPP Central	NEVP, PACE, IPCO, PSCO
NWPP NE	WACM, NWMT, WAUW, PACE
NWPP NW	PSEI, DOPD, CHPD, AVA, TPWR, GCPD, BPAT, PGE, PACW, SCL

Table A.4: Sub-region – balancing authority mapping to obtain aggregate demand

2.6.2 Generator Fleet

Initial Generator Fleet To construct our 2020 initial representative existing generator fleet, we begin with unit-level data on active existing units from *The National Electric Energy Data System* (NEEDS) dataset version 6 (updated in June 2020) (accessed 10/02/2021) [7]. Because NEEDS lacks storage unit parameters and other parameters need in our CE model, we merge the NEEDS dataset with EIA860 dataset [8] and add carbon dioxide (CO_2) emission rates from the the U.S. Energy Information Administration (EIA)'s *Carbon Dioxide Emissions Coefficients* [9], fuel prices from EIA's *Annual Energy Outlook 2020, Table 3. Energy Prices by Sector and Source* [10], and variable operation and maintenance (O&M) costs from [11]. We isolate generators within WECC,

our study region, using shape files of balancing areas within WECC from NREL's ReEDS model [12]. Our initial generator fleet is described in the table A.5. The *other* type of generators in the table below include geothermal, different types of waste, biomass, and other small fossil generators, which are all modeled as dispatchable capacity in the CEM and RAM.

Sub-region	Combined cycle gas	Simple cycle gas	Hydro	Nuclear	Steam turbine coal	Solar	Storage	Wind	Other
CAMX	20641	10825	10147	0	17	10644	3660	5764	4010
Desert Southwest	11256	4855	3840	3937	5333	2303	287	1488	363
NWPP Central	10486	5053	954	0	6693	3128	670	3636	1045
NWPP NE	94	465	3493	0	6562	40	0	2906	23
NWPP NW	6619	1669	32091	1180	0	356	364	6568	557

Table A.5: Initial generator fleet capacity of each generator type (in MW) across the subregions

Hydropower Generation In the CEM, we dispatch hydropower generation on a regional hourly basis as an energy-limited resource [ref eq. B.22]. Energy limits are defined for each time block using historic, weather-year-specific generation from Form EIA-923. We estimate monthly historic generation for each weather year by matching hydropower ORIS plant codes between our initial generator fleet and Form EIA-923. We then convert monthly generation to a total energy budget for each time block modeled in the CEM (4 representative blocks per season and 1 day for peak annual demand, net demand, and 1-hour upward ramp). This conversion happens in two steps. First, we divide monthly to hourly hydropower generation budgets using the proportion of monthly to hourly net demand. In some cases, this results in hours with generation exceeding regional hydropower capacity. For these hours, we iteratively reallocate surplus hourly generation to other hours using the proportion of monthly to hourly net demand, until regional hydropower generation does not exceed regional capacity in any hour. Finally, we sum hourly hydropower generation for all hours included in each time block.

Generator Fleet Compression Because the existing generation fleet in WECC is large with over 4,500 units, we combine (or aggregate) existing small generators into larger generators for computational tractability. We aggregate generators within the same region using two steps and several criteria. First, for each fuel type and plant type with zero marginal costs, we aggregate all generators into a single generator by region. Zero marginal cost generators include all geothermal, wind, solar, landfill gas, municipal solid waste, biomass, and non-fossil waste generators. Second, for each fuel type and plant type with non-zero marginal costs, we aggregate generators based on age and heat rate to preserve heterogeneity in operational costs. These non-zero marginal cost units include distillate fuel oil, natural gas combined cycle, natural gas combustion turbine, residual fuel oil, and coal (including bituminous, sub-bituminous, and lignite) generators. Specifically, by region, plant type, and fuel type, we divide generators into 4 heat rate blocks, then aggregate generators together within each heat rate block by decade between 1975 and 2026. We aggregate generators up to 200 MW in size in this manner, and create combined generators of up to 10,000 MW. These size thresholds significantly reduce the size of the generator fleet while still individually modeling mid- to large-sized power plants. Heat rates and CO_2 emission rates of the aggregated generators equal the capacity-weighted heat rates and CO_2 emission rates of their constituent generators.

2.6.3 Generator Investment Options

The CE model determines generator additions of three plant types: wind, solar PV, and 4-hour utility-scale battery storage. We obtain overnight capital costs and fixed and variable operation and maintenance (O&M) costs from NREL's Annual Technology Baseline (ATB) moderate technology development scenario for 2030 [11]. For computational tractability, we remove the lowest 40% of possible wind and solar investment locations in each region (i.e., grid cells) based on average annual capacity factor prior to running our CEM, leaving us with roughly 3,000 wind & solar locations across WECC.

2.6.4 System Topology

Our resource adequacy (RA) model uses the five regions that WECC uses to quantify resource adequacy in WECC [13]: NWPP NW, NWPP NE, CAMX, Desert Southwest, and NWPP Central [see figure A.3]. To align

regions between the CE and RA models, we model these same five regions in our CE model.

Within each of these regions, we ignore transmission constraints. Between regions, we enforce transmission constraints. Given the lack of data regarding transmission constraints between our WECC resource adequacy regions, we estimate inter-regional transmission constraints using data from the National Renewable Energy Laboratory (NREL) Regional Energy Deployment System (ReEDS) model. ReEDS provides transmission constraints between 35 balancing areas across WECC. We assign each balancing area to a region using spatial overlays, then set transmission constraints between each pair of regions as the sum of transmission constraints between each pair of balancing areas within each region. Using this method, we identify seven inter-regional, bi-directional transmission constraints. For each of these seven inter-regional transmission constraints, we limit hourly inter-regional electricity transfers to an upper capacity bound.

In addition to enforcing existing transmission constraints, the CE model can also invest in new transmission capacity between each of the seven inter-regional transmission interfaces identified above. Similar to other macro-scale planning models [14], we assume costs scale linearly with new transmission capacity, allowing us to maintain a computationally tractable linear program (LP). Per-MW costs of transmission expansion equal the distance (in miles) between the two centroids of interconnected regions times the per MW-mile cost of each bi-directional transmission line. We estimate this cost as the median of costs between each pair of balancing authorities between regions, which is taken from NREL's ReEDS Model's open access github [12]. Table A.6 depicts all possible combinations of aggregate links between our five load regions and their respective aggregate capacities and total cost per MW.

2.7 WECC subregions

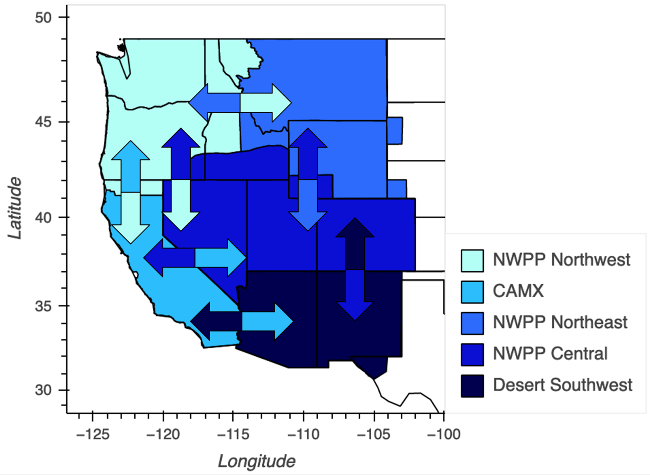

Figure A.3: WECC subregions used in the CEM and RAM. Arrows show transmission flows between the subregions.

Transmission Capacity between	Total Capacity (GW)	Expansion Cost (1000$/MW)
NWPP-NW and NWPP-NE	12.3	474
NWPP-NW and CAMX	7.1	1,018
NWPP-NW and NWPP-Central	1.5	569
NWPP-NE and NWPP-Central	6.0	431
CAMX and Desert Southwest	3.0	1,070
CAMX and NWPP-Central	4.6	816
Desert Southwest and NWPP-Central	5.6	348

Table A.6: Transmission Networks within WECC

2.8 Model Code and Data Availability

CEM code and data are available at `https://github.com/atpham88/US-CE`.

3 Resource adequacy model

3.1 Transmission between sub-regions

The transmission energy balance between the WECC subregions is modelled as a simple network flow problem without accounting for direction of flow in the circuit. For each iteration and each hour where there is a deficit in any sub-region, this flow problem is solved as a linear program.

3.1.1 Objective

The objective is to minimize the cost of transmission flow and cost of energy not served.

$$\min_{f,ens} \sum_{i} [T_c \times (\sum_{j,j\neq i} f_{ij}) + ENS_c \times e_i] \quad \forall i,j \in [1,N] \tag{C.29}$$

Where N is the number of sub-regions (henceforth referred to as nodes), f_{ij} is the unidirectional flow from node i to j, and e_i is energy not served or energy deficit at each node, T_c and ENS_c are the line transmission and energy not served cost (both $\$/MWh$).

3.1.2 Constraints

$$\begin{aligned} &\sum_{j,j\neq i} (f_{ij} - f_{ji}) - e_i \leq R_i \quad \forall i \in [1,N] \\ &0 \leq f_{ij} \leq F_{ij}, \quad e_i \geq 0 \quad \forall i,j \in [1,N] \end{aligned} \tag{C.30}$$

where $R_i \in \mathbb{R}$ is the residual or net load in each node and F_{ij} is the flow limits on each transmission line. When the residual is positive the node can export and when the residual is negative the transfers into the region is positive or there is unserved energy.

3.2 RAM iteration convergence

Figure A.4 provides the LOLH, EUE, and simulation time across 25 simulations for 250 and 500 Monte Carlo iterations for the weather year 2017 and 45% RE penetration scenario. As the iteration size increases, the distribution of LOLH estimates tightens. Increasing iterations results in narrowing of the LOLH distribution, with similar range in EUE, but increases computation time by more than 3x. For other weather years and RE scenarios, the simulation times is much higher, for instance, with RE=45% and 2019 weather year, this simulation takes around 4 hours to complete. Since we are more interested in the timing of the risk hours and not amount of risk throughout our analysis, this variation in LOLH does not impact our findings.

3.3 Forced outage rate

Table A.7 shows the outage probabilities of the various generators as a function of ambient temperature.

Table A.7: Temperature dependent forced outage rates of different generators

Closest temperature value [$^\circ C$]	-15	-10	-5	0	5	10	15	20	25	30	35
Nuclear	1.9 %	1.8 %	1.7 %	1.8 %	1.9 %	2.1 %	2.7 %	3.1 %	3.9 %	6.6 %	12.4 %
Combined cycle gas	14.9 %	8.1 %	4.8 %	3.3 %	2.7 %	2.5 %	2.8 %	3.5 %	3.5 %	4.1 %	7.2 %
Simple cycle gas	19.9 %	9.9 %	5.1 %	3.1 %	2.4 %	2.2 %	2.4 %	2.7 %	3.1 %	3.9 %	6.6 %
Steam turbine coal	13.3 %	11.2 %	9.9 %	9.1 %	8.6 %	8.3 %	8.4 %	8.6 %	9.4 %	11.4 %	14. %
Hydro	7 %	4.3 %	3.2 %	2.7 %	2.6 %	2.6 %	2.7 %	2.7 %	2.5 %	2.9 %	8.2 %
Solar, wind, storage, other	5 %	5 %	5 %	5 %	5 %	5 %	5 %	5 %	5 %	5 %	5 %

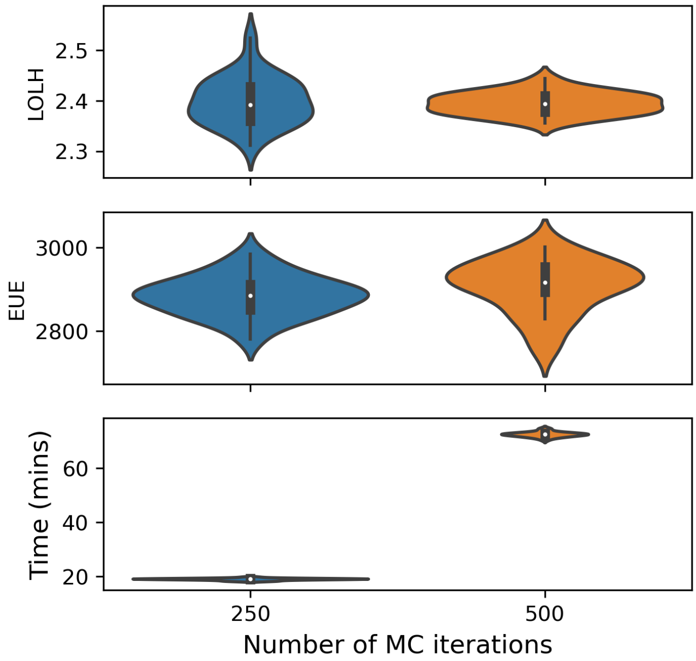

Figure A.4: Variation in range of LOLH with increasing number of Monte Carlo samples

4 Self organizing maps and Weather Patterns

To test the sensitivity of the SOM technique to grid size and training iterations for identifying weather regimes (WR), we use the metrics quantization error (QE) and topographic error (TE) [15]. QE represents the variance within the SOM node and is calculated as the L2 error between the daily circulation maps assigned to a node and the node centroid. TE represents the continuity in the map. TE is calculated by finding the fraction of inputs for which the best matching node (the node it is assigned to) and the second best matching node are not neighboring WRs. So, we want to minimize QE to make the node centroid (weather pattern for our purposes) more representative of the maps assigned to it and minimize TE to ensure the map nodes are topologically continuous. Figure A.5a shows how QE and TE vary for different grid shapes used to train the SOM. We find that a 3x3 grid produces a map that best balances QE and TE. Figure A.5b shows the sensitivity of QE and TE to training iterations. We find 1000 or 5000 iterations is optimal to minimize both QE and TE. Though 1000 iterations does marginally better in comparison to 5000 iterations, we get more stable maps when retraining using 5000 iterations, hence use that to obtain our weather patters.

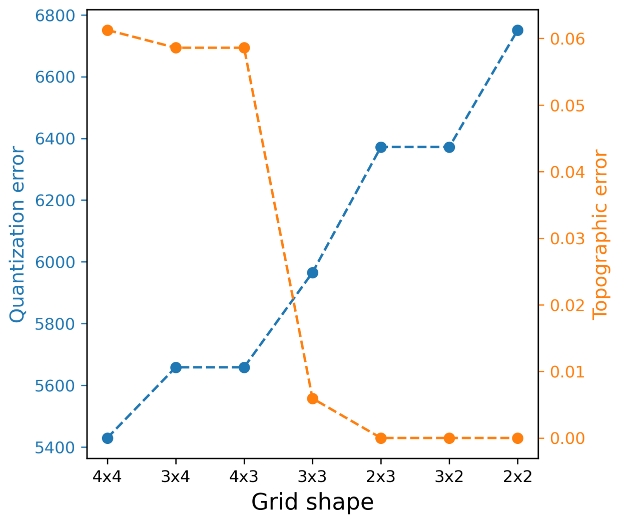

(a)

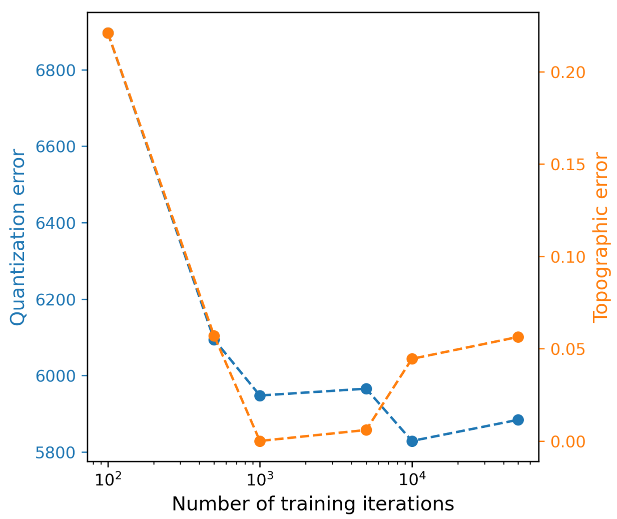

(b)

Figure A.5: Quantization and topographic error for different (a) grid shapes of the SOM (row x columns) (b) training iterations

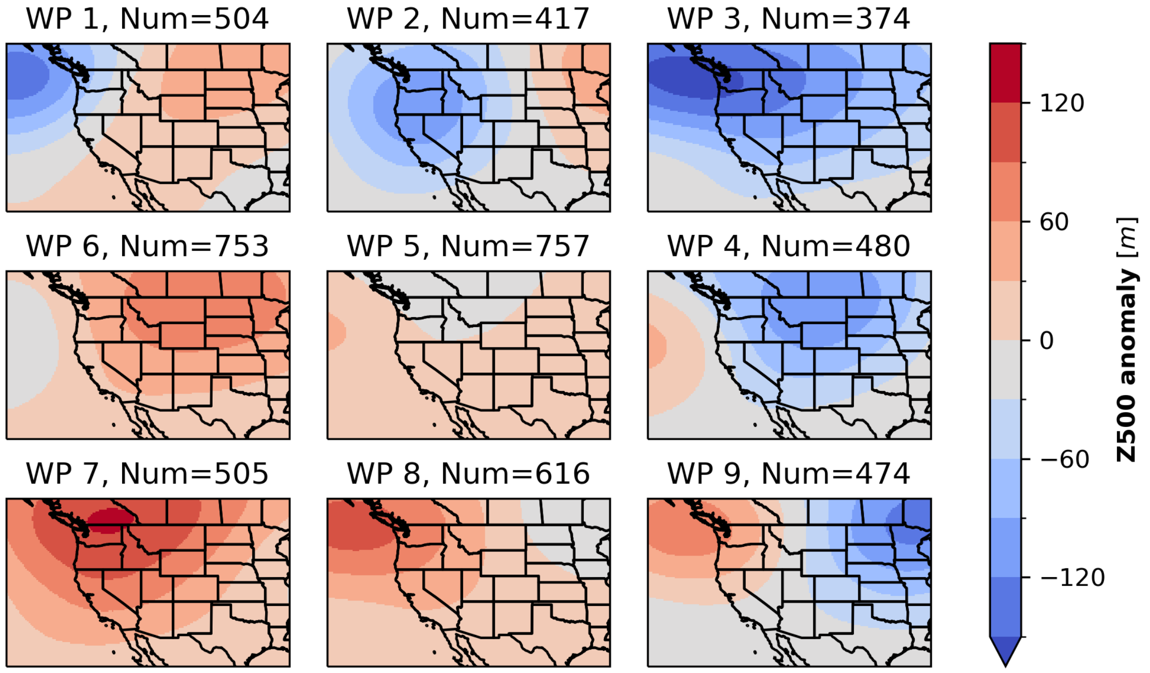

Figure A.6: Weather patterns representing the weather regimes with the titles for each panel indicating the number of extended summer days from June-September from 1981-2020 that fall into each weather regime

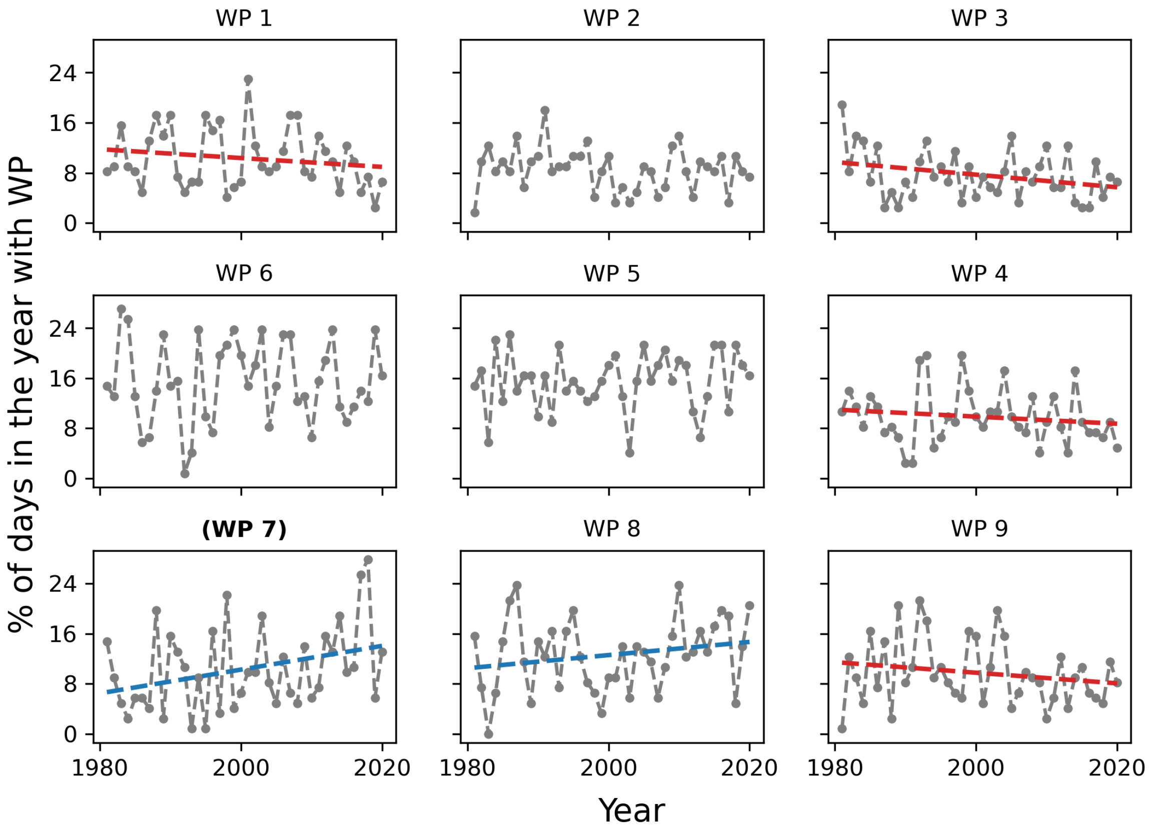

Figure A.7: Grey dots show the percentage of extended summer days from 1981 - 2020 belonging to each weather regime. Red (negative slope) and blue (positive slope) dotted lines show a linear regression if the trend is greater than or equal to —0.05— and bold parenthesized text indicates a 95% statistical significance of regression coefficient

5 Results SI

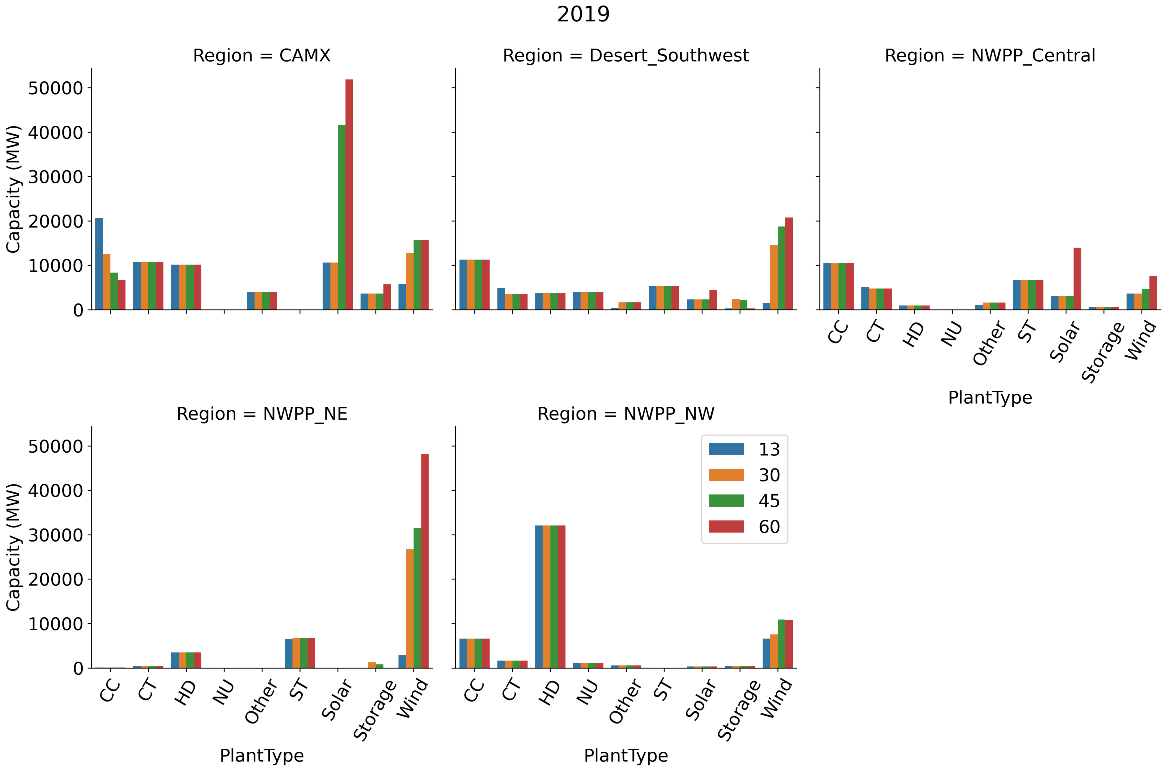

Figure A.8: For the 2019 weather year this figure shows installed capacities of different generation sources in the subregios with increasing renewable penetrations.

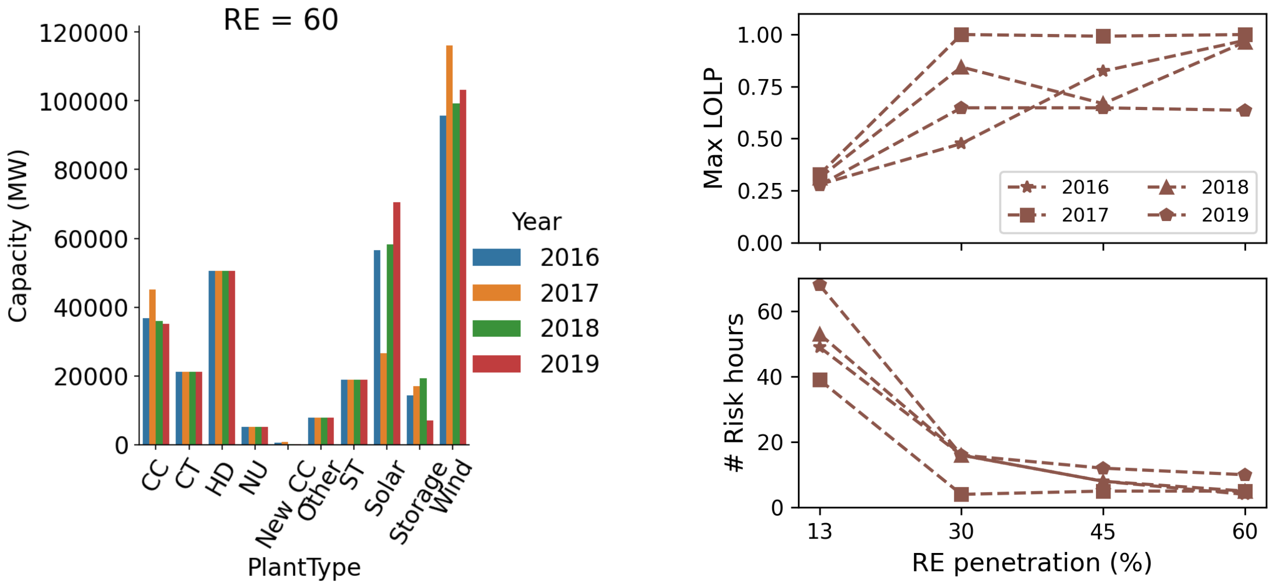

(a)

(b)

Figure A.9: (a) Installed capacity of different generation assets across the weather years with at 60% RE penetration; (b) Max LOLP (top) and number of risk hours (bottom) across the weather years with increasing RE generation levels;

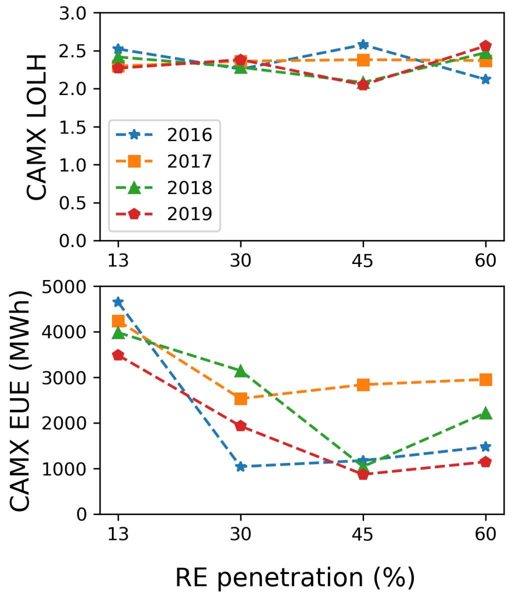

Figure A.10: LOLH and EUE across the weather years with increasing RE generation levels

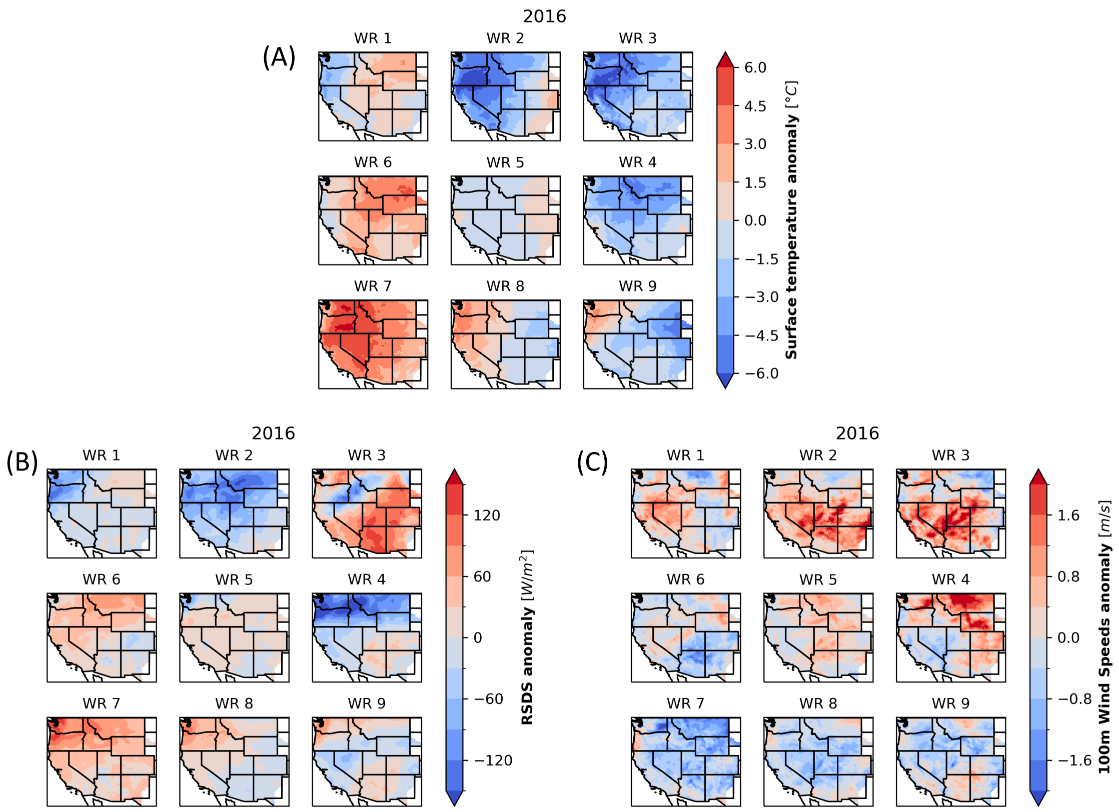

Figure A.11: Composites of surface temperature (A), surface solar radiation (B), and 100m wind speeds (C) anomalies. The composites are constructed based on the hours from 2016 extended summer belonging to each weather regime.

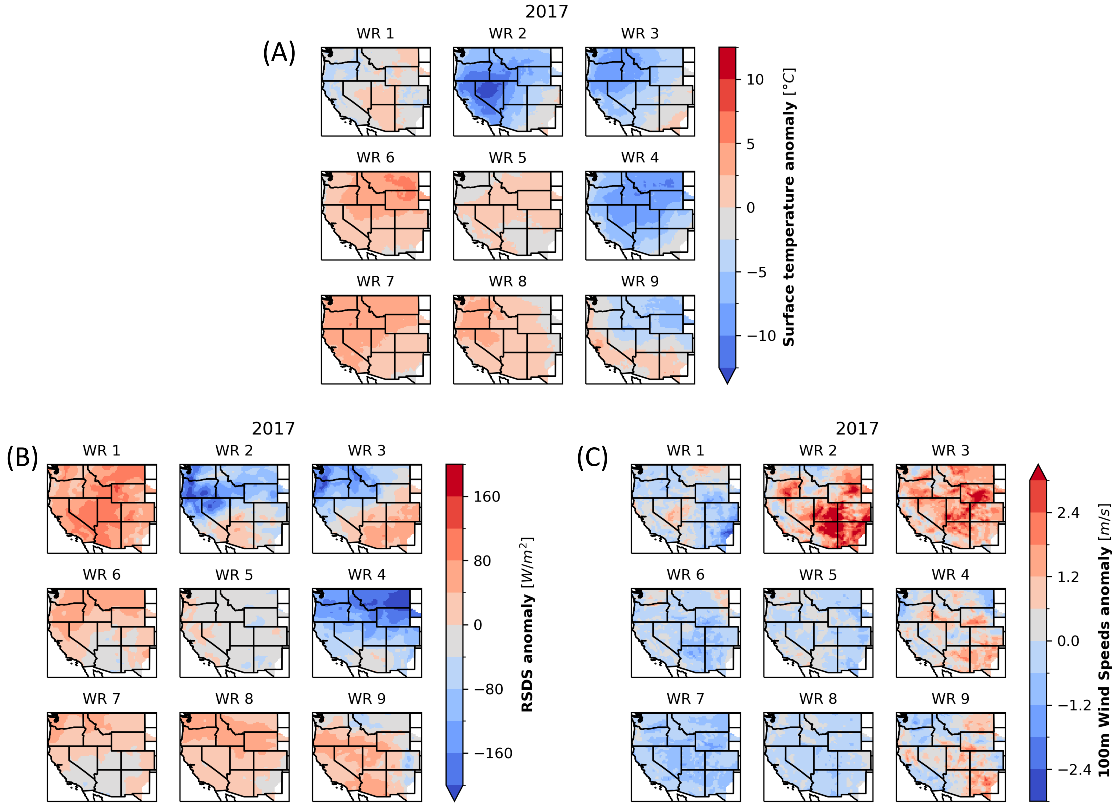

Figure A.12: Composites of surface temperature (A), surface solar radiation (B), and 100m wind speeds (C) anomalies. The composites are constructed based on the hours from 2017 extended summer belonging to each weather regime.

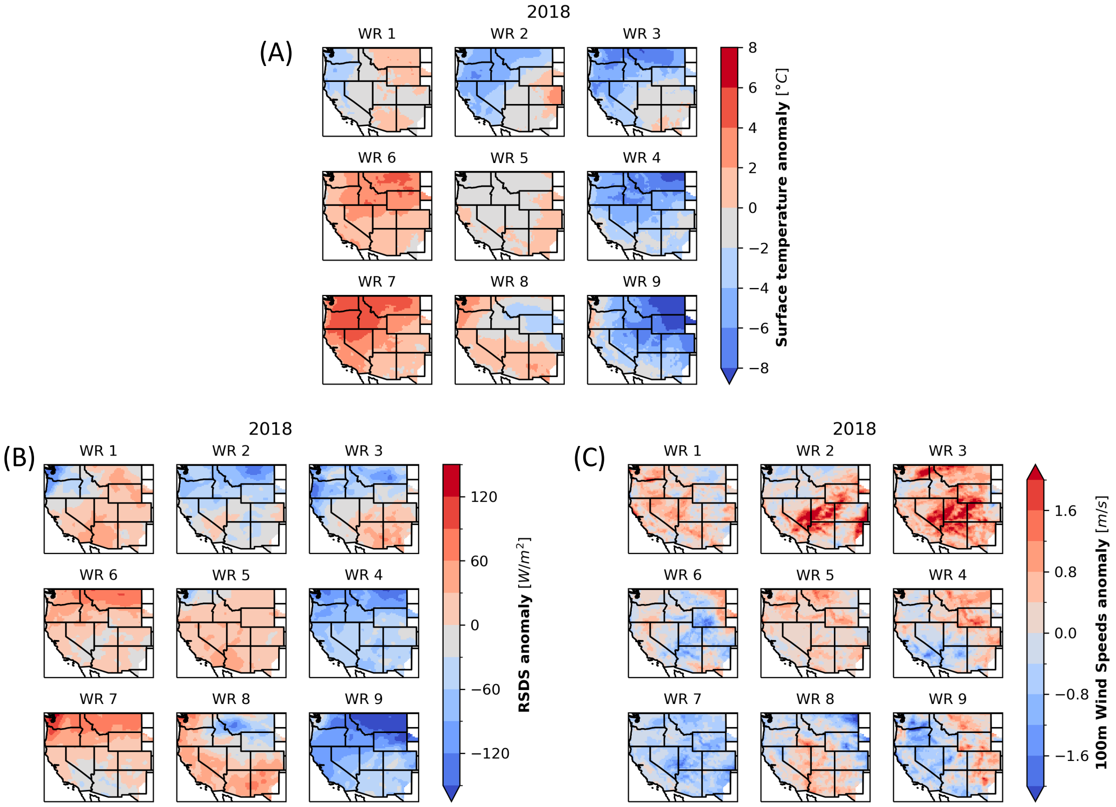

Figure A.13: Composites of surface temperature (A), surface solar radiation (B), and 100m wind speeds (C) anomalies. The composites are constructed based on the hours from 2018 extended summer belonging to each weather regime.

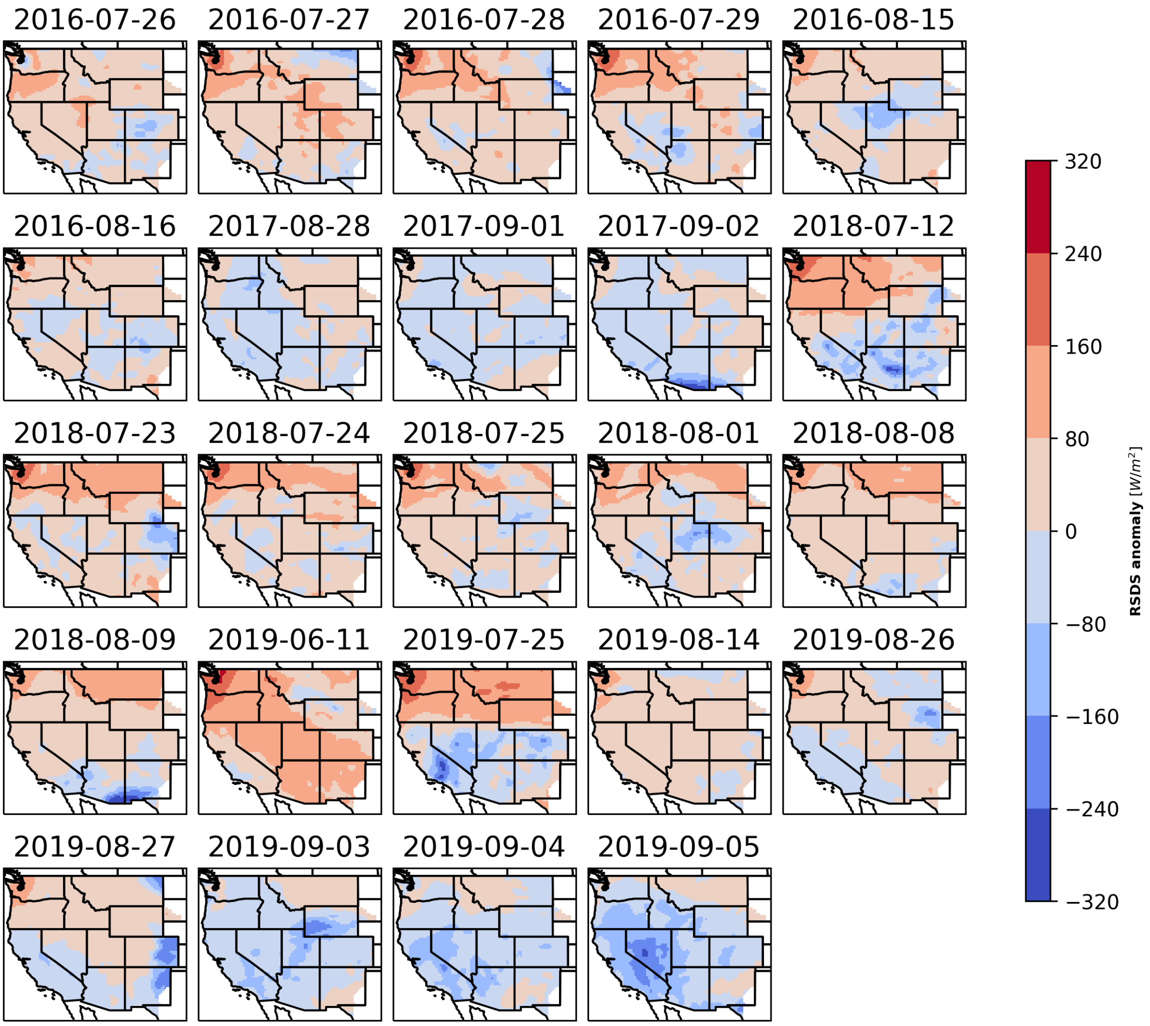

Figure A.14: Daily surface solar radiation anomalies on days with RA failure events for RE penetrations from 30% to 60% across the weather years.

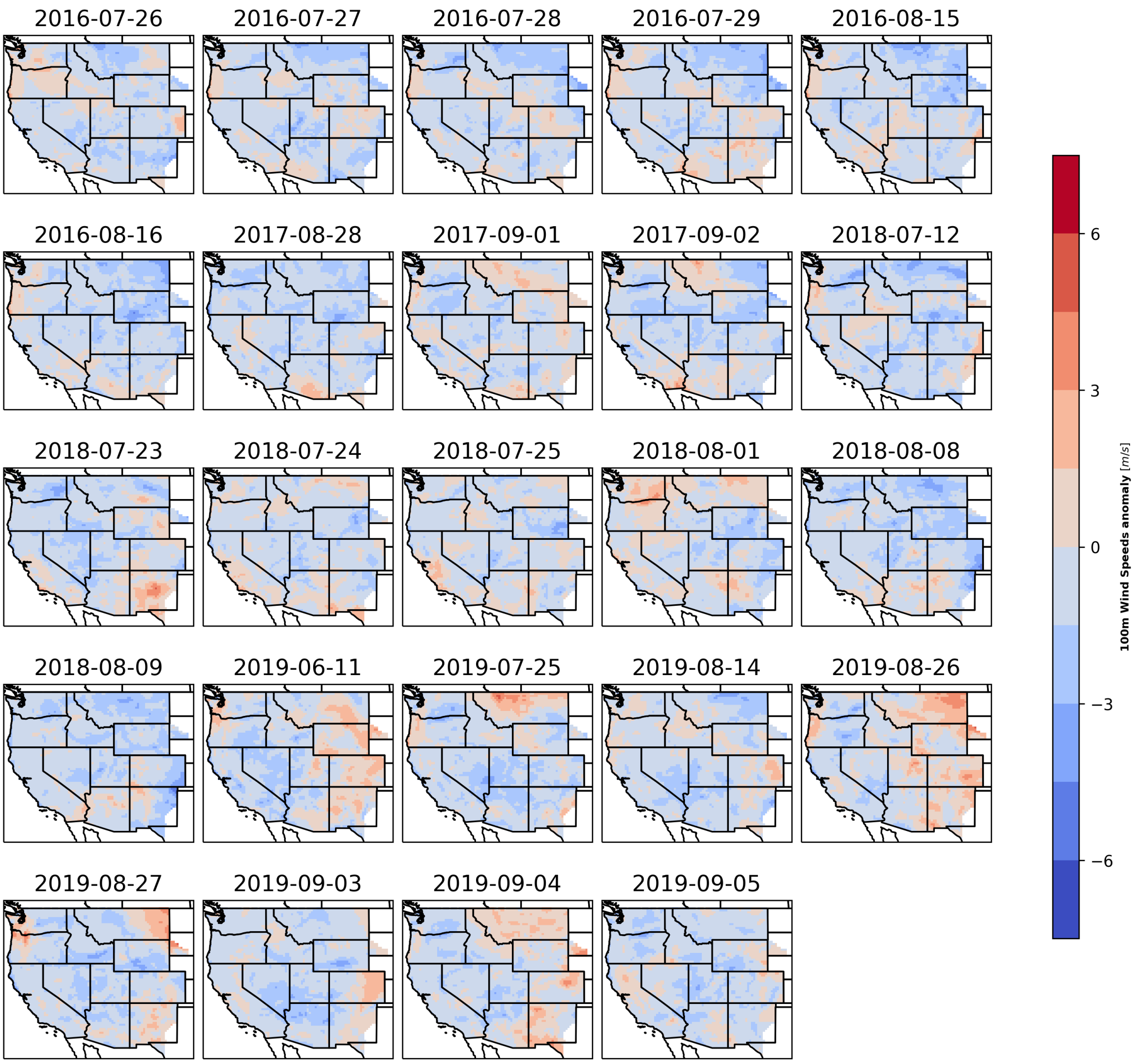

Figure A.15: Daily 100m wind speeds anomalies on days with RA failure events for RE penetrations from 30% to 60% across the weather years.

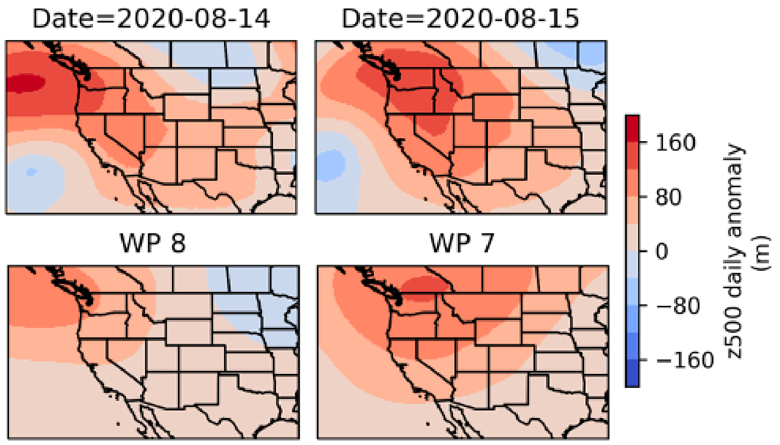

Figure A.16: Daily Z500 anomaly on August 14th and 15th 2020 (Top panels) and WPs 8 and 9 from the extended summer weather regimes (Bottom panels).

References

[1] Sonia Jerez, Isabelle Tobin, Robert Vautard, Juan Pedro Montávez, Jose María López-Romero, Françoise Thais, Blanka Bartok, Ole Bøssing Christensen, Augustin Colette, Michel Déqué, et al. The impact of climate change on photovoltaic power generation in europe. *Nature communications*, 6(1):1–8, 2015.

[2] Govindasamy TamizhMani, Liang Ji, Yingtang Tang, Luis Petacci, and Carl Osterwald. Photovoltaic module thermal/wind performance: long-term monitoring and model development for energy rating. In *NCPV and Solar Program Review Meeting Proceedings, 24-26 March 2003, Denver, Colorado (CD-ROM)*, number NREL/CP-520-35645. National Renewable Energy Lab., Golden, CO.(US), 2003.

[3] Kristopher B Karnauskas, Julie K Lundquist, and Lei Zhang. Southward shift of the global wind energy resource under high carbon dioxide emissions. *Nature Geoscience*, 11(1):38–43, 2018.

[4] Nate Blair, Nicholas Diorio, Janine Freeman, Paul Gilman, Steven Janzou, Ty Neises, and Michael Wagner. System advisor model (sam) general description (version 2017.9. 5). *National Renewable Energy Laboratory Technical Report*, 2018.

[5] David Bolton. The computation of equivalent potential temperature. *Monthly weather review*, 108(7):1046–1053, 1980.

[6] Lee M Miller and David W Keith. Observation-based solar and wind power capacity factors and power densities. *Environmental Research Letters*, 13(10):104008, 2018.

[7] EPA. U.S. Environmental Protection Agency 2020 National Electric Energy Data System (Version 6.0). Online, 2020.

[8] Bipartisan Policy Center. Form eia-860 detailed data with previous form data (eia-860a/860b). *Energy Information Administration, Washington, DC*, 2020.

[9] EIA. Carbon Dioxide Emissions Coefficients. Online, 2020.

[10] AEO. Annual Energy Outlook 2020. Online, 2020.

[11] Sertaç Akar, Philipp Beiter, Wesley Cole, David Feldman, Parthiv Kurup, Eric Lantz, Robert Margolis, Debo Oladosu, Tyler Stehly, Gregory Rhodes, et al. 2020 annual technology baseline (atb) cost and performance data for electricity generation technologies. Technical report, National Renewable Energy Laboratory-Data (NREL-DATA), Golden, CO (United …, 2020.

[12] NREL. ReEDS OpenAccess. github, 2020.

[13] WECC. Western assessment of resource adequacy, Nov 2022.

[14] Jesse D Jenkins and Nestor A Sepulveda. Enhanced decision support for a changing electricity landscape: the genx configurable electricity resource capacity expansion model. 2017.

[15] Cassandra DW Rogers, Kai Kornhuber, Sarah E Perkins-Kirkpatrick, Paul C Loikith, and Deepti Singh. Sixfold increase in historical northern hemisphere concurrent large heatwaves driven by warming and changing atmospheric circulations. *Journal of Climate*, 35(3):1063–1078, 2022.
